# It’s Not Really the Words; It’s How They Land: Lived Experience Perspectives on the Use of Language and Labels in Mental Health

**DOI:** 10.3390/ijerph23081032

**Published:** 2026-08-07

**Authors:** Anna Foxcroft, Craig Allen, Julia Dray, Amelia Gulliver, Dianna Smith, Grenville Rose, Bridget Berry, Michelle Banfield

**Affiliations:** 1 Centre for Mental Health Research, National Centre for Epidemiology and Population Health, The Australian National University, Canberra, ACT 2601, Australia; anna.foxcroft@anu.edu.au (A.F.); craig@inspiredmindset.com.au (C.A.); julia.dray@uts.edu.au (J.D.); amelia.gulliver@anu.edu.au (A.G.); dianna.smith@anu.edu.au (D.S.); grenville.rose@anu.edu.au (G.R.); bridget.berry@sydney.edu.au (B.B.); 2 The ALIVE National Centre for Mental Health Research Translation, The Australian National University, Canberra, ACT 2601, Australia; 3 Graduate School of Health, Faculty of Health, University of Technology Sydney, Sydney, NSW 2007, Australia

**Keywords:** language, labels, lived experience, mental health, stigma, identity, communication

## Abstract

**Highlights:**

**Public health relevance—How does this work relate to a public health issue?**

- Mental health is a key part of a holistic approach to public health.
- The way we describe mental health and ill-health plays an important role in health promotion.

**Public health significance—Why is this work of significance to public health?**

- Language is a key factor across multiple levels of health determinants, operating structurally, as an influence on knowledge, attitudes and beliefs.
- At an individual level, language shapes the control people feel over their health and can have opposing influences.

**Public health implications—What are the key implications or messages for practitioners, policy makers and/or researchers in public health?**

- A genuinely recovery-oriented system needs to prioritise non-stigmatising and inclusive language in policy, practice and research.
- Careful attention to personalised communication and seeing people beyond their labels may improve health behaviours and public attitudes.

**Abstract:**

The language used to describe and discuss mental ill-health continues to evolve and improve, driven by advocacy and commitment to reducing the harmful effects of stigma. However, from a lived experience perspective, room for improvement remains. This project aimed to explore the use of language and labels within the mental health sector and its impact on people with lived experience of mental ill-health. Interviews were conducted with mental health consumers and/or carers (*n* = 15), and co-researchers with lived experience of mental ill-health contributed researcher positionality to the research. Six themes were generated using reflexive thematic analysis; they accented how participants expressed complex and nuanced experiences of language and labels, and the overlapping themes highlight the ‘blurry edges’ when people with lived experience discuss language, with the individual at the centre. Responses reflected the nature of inclusive and exclusive language in the medical system and the community. Central to experiences of exclusion were times when people felt dismissed and misunderstood due to the language and labels others use and how the words are delivered. These negative experiences reflect the influence of stigma. Positive language provides validation, understanding and a sense of hope. Health professionals may have opportunities to apply more personalised communication approaches, which could improve outcomes for the people they support.

## 1. Introduction

The language used to describe mental ill-health has long been a contentious issue, contributing to poor public attitudes and beliefs [1]. Prior to the 20th century, terms such as ‘lunatic’, based on the theory that the moon exacerbated distress, were common [1]. Later words like ‘mad’ or ‘insane’ appeared along with the creation of ‘asylums’, which, despite implying refuge, often resembled prisons, with residents called ‘inmates’ [2]. By the late 1800s, ‘mental disease’ gained popularity, marked by the advent of the world’s oldest scientific monthly journal on the study of mental health, The Journal of Nervous and Mental Disease, established in 1874 [3]. In the 1900s, medicalised language began to emerge, such as ‘mental hospitals’ and ‘patients’ [1]. The 1950s introduced ‘mental disorder’, reflecting new diagnostic categories described in the first version of the Diagnostic and Statistical Manual of Mental Disorders (DSM-I) [4]. Since then, terms including mental ‘illness’, ‘problem’, ‘issue’ and ‘condition’ have all become common [5]. More recent terminology includes the use of ‘mental-ill health’ [6], which is adopted in the current paper as an umbrella term.

Research shows language contributes to harm, distress and stigma for people with mental ill-health [7], which may undermine efforts to improve help-seeking and a sense of control over health. Certain words carry more perceived stigma than others when it comes to describing experiences related to mental ill-health [8]. Many terms such as ‘lunatic’ remain stigmatising, whilst others are being reclaimed by people with lived experience, e.g., ‘mad’ [9]. Thus, stigmatisation of words depends on the meaning behind them and how they are used. Labelling, and the separation of ‘us’ from ‘them’ [10], has contributed to stigma in health care; for example, the use of ‘frequent flyer’ for those who are perceived to access health services often [11]. Stigmatising terms are also used in other settings including research [12], news and social media [13].

Which words are considered stigmatising remains an evolving topic. Person-first language is common in physical ill-health (e.g., a person ‘has cancer’), reinforcing inclusion [14], whereas mental ill-health historically lacked this inclusive approach to language (e.g., ‘schizophrenic’) [10]. The adoption of person-first language marked progress towards reducing stigma around mental ill-health [15], yet challenges persist, including inconsistent use in health care settings, which has been identified as a problem impacting the integration of peer workers into health care teams [16]. However, person-first language preference is not universal across all identities and conditions. For example, people who are neurodivergent often favour identity-first language (i.e., ‘autistic’) to affirm identity, rather than ‘person with autism’ [17]. Personal preferences also exist, determined by setting, individual, and cultural preferences [18], with individual preferences for terms like ‘patient’, ‘client’, ‘consumer’, ‘service user’ [19,20] or ‘person with lived experience’. These variations highlight the need for continued open communication and review of language both in individual care encounters and at a broader systemic level.

ACACIA: The ACT Consumer and Carer Mental Health Research Unit at The Australian National University (ANU) was established in 2013 to undertake mental health research inclusive of and relevant to the Australian Capital Territory (ACT) mental health sector. Lived experience researchers partner with consumers and carers to research lived experience priorities and build consumer and carer research capacity [21,22]. The project *What we call ourselves: exploring the use of language in mental health* was developed from a lived experience research agenda topic [21]. Thus, the aim of this research was to explore the use of language and labels within the mental health sector and its impact on people with lived experience of mental ill-health. Previous research has broadly examined language and stigma [12,13,14] and the impacts of person-first language [15,17]. However, our interest was specifically to investigate, from a lived experience perspective, how various terms used to address or describe people with mental ill-health may affect experiences of care, health-promoting behaviours and social inclusion, which represented a current gap in the literature.

We have chosen to use the terms consumer, carer and lived experience throughout this paper as these are the terms commonly used in Australia. We acknowledge that many of our participants did not like or use these terms to describe themselves, as they felt the terms failed to convey their experience, fully describe them as a whole person or understand them as an individual. While the cohort of participants who represented carers in this research did use the carer term, we recognise that not all people performing this important role use the term. In this paper, the term carer is used for readability, instead of the longer and more inclusive expression “carer, family and kinship group members”.

## 2. Materials and Methods

### 2.1. Ethics Approval

The ethical aspects of this project were approved by the ANU Human Research Ethics Committee, Protocol 2022/753.

### 2.2. Lived Experience and Positionality

Embedding lived experience into this research is consistent with all research undertaken at ACACIA [22]. Staff and students at ACACIA are mental health consumers or carers with academic expertise and qualifications [22]. The research questions explored in the project were created during lived experience priority setting co-design activities and are relevant to carers and consumers [21]. The ACACIA Advisory Group [22] co-developed the research protocols and materials, ensuring information and interview questions were appropriate for participants with lived experience of mental ill-health. Lived experience researchers at ACACIA provided project management and mentorship, including support to recruit and manage participants, team meetings and research guidance through the data collection, analysis and write-up stages.

The research was conducted as part of an internship programme, running from March to October 2023. Two co-researchers (authors A.F., female and C.A., male) were competitively recruited as research interns with lived experience of mental ill-health as either a consumer or carer and without an academic research background. The lived experience co-researchers were embedded into the research team, and received skills training developed by ACACIA including ethical research, interview techniques, qualitative data analysis and academic writing. ACACIA’s short courses focused on recognising and using the co-researchers’ lived experiences to connect with participants and engage with the data [23,24]. The co-researchers were encouraged to disclose their lived experience and the perspective they brought to the research as part of the interview introduction [23,25].

### 2.3. Procedure

#### 2.3.1. Participants and Recruitment

Participants who identified as mental health consumers and/or carers over the age of 18 were recruited by an invitation circulated to the ACACIA emailing list, a database of people who have expressed interest in ACACIA’s research. Invitations were also emailed to members of The ALIVE National Centre for Mental Health Research Translation network (ALIVE) who expressed interest in the project following an ACACIA presentation at an ALIVE symposium in March 2023. Following distribution of recruitment material, 24 people asked to receive participant information. Fifteen people chose to be part of the project; all participants provided written informed consent and received an AU$30 gift voucher for their participation. Carers were encouraged to discuss their participation with the person they care for before their interview, and not to divulge sensitive personal or health information about other people during the interview [26].

Participants were emailed a copy of the results section of this paper prior to submission and invited to provide advice if they wanted any comments they recognised as their own removed from the manuscript; however, no formal feedback was provided.

#### 2.3.2. Data Collection

Participants were provided with the option to attend interviews face-to-face, by phone or video conference. Fifteen interviews were conducted, 12 via Zoom video conferencing (Zoom Video Communications Inc., San Jose, CA, USA) and three in person at the ACACIA office on the ANU campus. All interviews were audio recorded, with participants’ consent, and transcribed by an external professional transcription service, subject to a confidentiality agreement. Interviews were conducted by authors A.F. and C.A. (intern co-researchers) and lasted 30–60 min. The interviews were semi-structured and participant-led, intending to hear from participants in their own words and provide a deep understanding of the personal and broader social impacts of language and labels for people with lived experience of mental ill-health.

The interviews were guided by two questions:

1. How do you think the use of language includes or excludes individuals?
2. What is your opinion on the use of labels, which terms are useful/helpful, which are not? *Prompt: diagnostic labels, client/consumer/patient/service user labels.*

We asked follow-up questions where necessary, to explore participant experiences in more depth. Participants also completed a brief demographic survey to enable a basic description of sample characteristics, comprising gender (self-described), age (by category), lived experience (consumer, carer, both) and Australian state/territory of residence.

#### 2.3.3. Data Analysis

This qualitative exploratory study used reflexive thematic analysis [24]. Reflexive thematic analysis (RTA) acknowledges the positionality of the researcher, and the “knowingness” that they bring to the research [24,27]. This approach enabled the co-researchers to draw on previous personal and professional experiences, combine these with the acquired research skills and apply their whole lived experience to the project to shape the interpretation of the data [23,28]. In addition to recognising the positionality of the researcher as a resource in RTA, Braun and Clarke [24] describe other benefits including meaning-making and storytelling. These aspects of RTA make it a powerful method for lived experience co-research where the intent is to tell stories from the participants’ perspectives and maintain their voice in the interpreted narrative [29,30].

Co-researchers (A.F. and C.A.) undertook training in RTA provided by the ACACIA team and reviewed online training materials [31]. To assist with the analysis, co-researchers kept interview field notes and wrote reflexive notes after each interview [32,33]. The co-researchers could debrief with another team member and discuss emerging thoughts during regular team meetings. These reflections enabled the co-researchers to explore observations and ideas with each other and further participants during subsequent interviews. This reflexive and iterative approach to interviews and analysis prioritised the depth and richness of data over notions of saturation through reflexive engagement during data collection and analysis [34]. However, we note the decision to end recruitment at 15 interviews was also pragmatic: the limited timeframe of the internship program together with the depth of information we had gathered at this point indicated that the data was sufficient to meet the study aims.

Demographic data were tabulated with percentages calculated for each characteristic. Australian Bureau of Statistics standard classifications [35] were used to categorise the participants’ self-described gender.

#### 2.3.4. Data Coding and Theme Development

Alongside data collection and to become familiar with the data [36], the co-researchers each listened to all interviews prior to initial coding. Each co-researcher checked accuracy and de-identified transcripts for the participant interviews they conducted. Using an inductive RTA approach, co-researchers leaned into their lived experience to enrich interpretation, using explicit and latent coding to identify patterns of meaning [24,37]. Both co-researchers undertook initial coding, starting with the participant interviews they each conducted, and to aid the ongoing reflexive dialogue and interpretive development, six interviews were coded by both co-researchers. Microsoft Word and Excel (Windows 10, Microsoft Corporation, Redmond, WA, USA) were used as tools for initial coding [38], alongside pen and paper techniques. These methods were encouraged as an opportunity for the co-researchers to engage with the data using their existing personal and professional skill sets. The co-researchers’ alternative insider perspectives [23,25] meant that they each identified and responded to different themes. Co-researcher 1 brought experience navigating systems as a family carer, and co-researcher 2 brought consumer experience in overlapping service systems. Both had experienced stigma and the personal effects of poor language and labelling. Initial coding by each co-researcher reflected resonance with experiences most similar to their own position. Collaboration between the co-researchers enabled a more nuanced understanding of participants’ stories. Ongoing reflexive discussion enabled the development of a shared understanding of the broader scope of experiences and how they could shape concepts and themes.

Following initial coding, the co-researchers had inductively created 133 codes reflecting concepts of mental health, lived experience, language, labels, communication and identity (see Table S1). The team (M.B., J.D., G.R., A.F., C.A.) met, giving the co-researchers an opportunity to discuss what they understood from the data and draw on the academic researchers’ expertise in qualitative analysis to develop themes. During this meeting, the co-researchers presented paper copies of the codes and the team worked together to group the codes and the concepts discussed into candidate themes. The initial analysis showed that people’s experiences of language and labels were complex and nuanced, with many of the themes overlapping. Early theme development identified six main themes, containing three overarching themes (people’s experiences are different, language evolves over time and power of positive and negative language; see Table S2). These initial themes were reflected in a draft findings report (unpublished) at the end of the internship. Themes were further analysed and refined by authors A.F. and M.B. to better reflect the interconnections and meaning [32,34]. As shown in Table 1, these concepts are explored in this paper through six overlapping themes: ‘how the words land’, feelings of inclusion and exclusion, labels and how they land, identity and connection, language evolves—who decides?, and doing things differently—understanding the person. The final framework centres the theme ‘how the words land’, which integrates the original three overarching themes and embraces the ‘blurry edges’ of language and labels with the individual at the centre.

## 3. Results

### 3.1. Participants

A total of 15 people with lived experience of mental ill-health participated in the study. As shown in Table 2, almost half the participants were consumers, a quarter were carers, and a quarter were both consumers and carers. Overall, 80% of participants were female, with all participants aged over 35 years and a relatively even spread across these age groups. The participants were located around Australia, with nearly three-quarters from the ACT or Victoria, reflecting the states in which ACACIA and ALIVE are based.

### 3.2. Findings

Almost all participants responded to the first interview question with an exclamation similar to “that is a big question” and, without any need for clarification or reframing, went on to answer the question from a variety of perspectives and in different ways. Carer participants provided insights and experiences about language and labels in relation to the people they care for and also described the impact of language on carers themselves, their identity and relationships. Where carer perspectives reflected a unique viewpoint, they are discussed separately; otherwise carer views are integrated into the findings.

**Figure 1:**
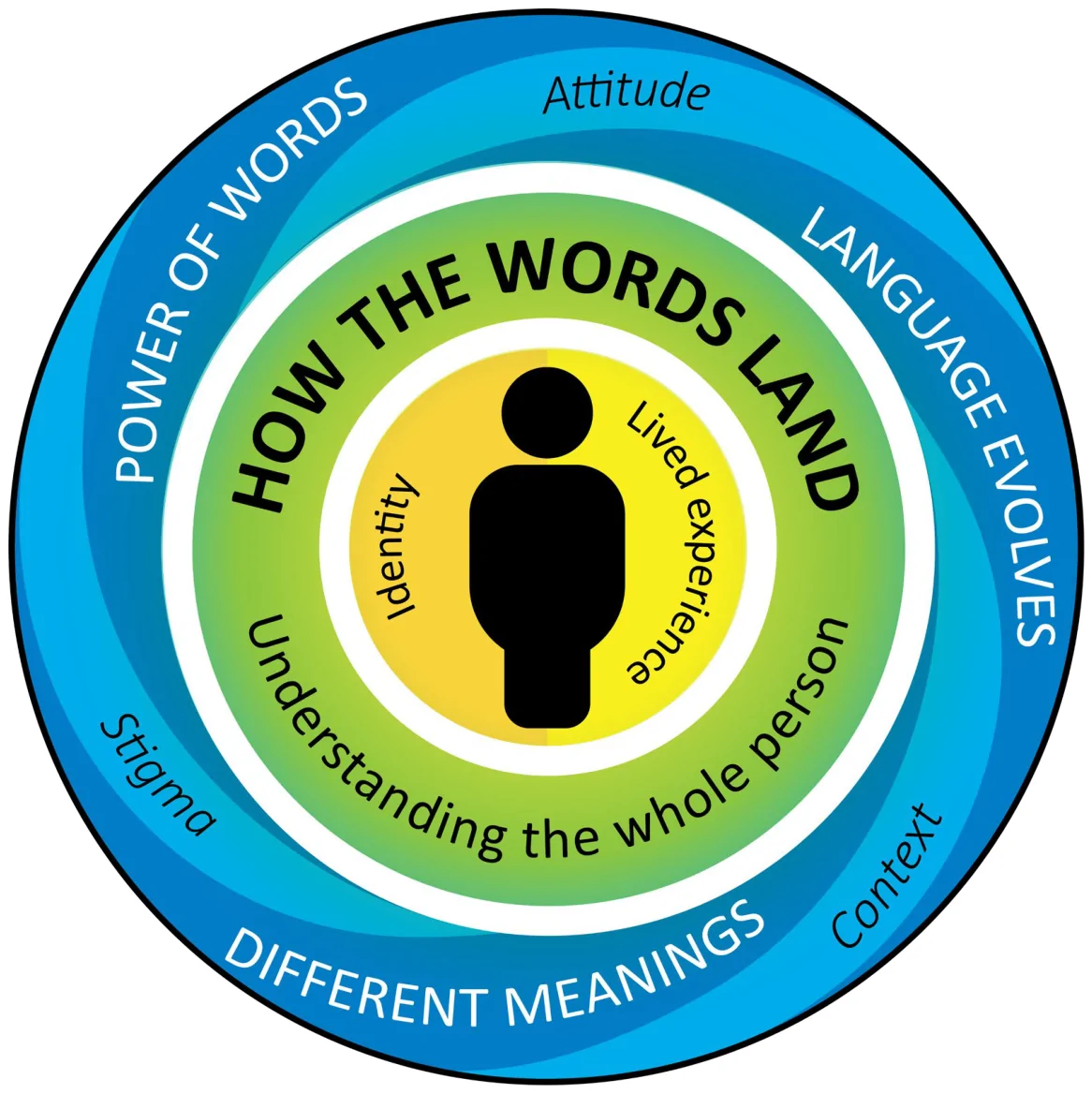
The relationship between themes—internal and external factors that shape personal experiences of language and labels.

The narrative presented in the paper surrounds the central theme ‘how the words land’, characterising the impact of language and labels on the individual. This central theme represents the intersection between the internal and external factors that shape personal experiences of language and labels. The framework in Figure 1 was developed to demonstrate the complex interrelation of language and labels in lived experience, and show how the themes overlap. From the outside, ‘how the words land’ is influenced by the power of words, different meanings attached to words and evolving language, which frame context and delivery (including stigma). From the inside, ‘how the words land’ depends on the individual, is connected to identity and importantly, ‘how the words land’ can lead to both positive (inclusive) and negative (exclusive) lived experiences. Responding to these concerns, participants provided ideas for doing things differently by respecting the individual and understanding the whole person.

#### 3.2.1. How the Words Land

Acknowledging everyone as an individual with unique experiences, for whom language is felt differently, is the heart of this central theme. Participants described different experiences and emotions attached to the language used and the labels applied to them and their journey, foregrounding the need to understand each person as an individual. Participants spoke about language in relation to experience, highlighting that although people may have the same label, their experiences and presentations can be completely different.

*“Everyone’s different, you know. It’s like two people can have the flu and they will understand that well, “Oh, OK, I’ve got the headache and the sore throat, oh and you don’t have either of those, but you’ve got aches and chills, and we’ve both got the flu, but our experience of it is completely different.”*

*(Participant 10, consumer)*

Conversations revealed that people use language to make sense of their own lived experiences, and that because of unique life journeys, background, and culture, words mean different things to different people and in different contexts. Participants expressed the difficulties this creates when talking about mental ill-health, especially as language is constantly evolving.

*“I think you can move the culture and the language, like we have with everything… And of course, language changes over time because it gets, it picks up derogatory meanings and then it has to change.”*

*(Participant 3, carer)*

Some of the ways participants acknowledged positive changes to language were through use of neutral language that does not make assumptions about anyone or anything, the importance of person-centred language, and using inclusive language.

*“It doesn’t happen so much these days, but some nurses would say in the handover, on the medical surgical wards as well as the psych wards, Oh “The hip replacement in room 10,” or, “The incontinence in room 8.” And it’s just like demeaning to people. So as most people know by now […] it’s like, oh the person with the broken leg. But it does make a big difference.”*

*(Participant 11, consumer and carer)*

Participants described the power of words and how language shapes our experiences: *“I think they [words] can be beautiful and they can be awful” (Participant 15, consumer and carer);* in some cases, giving us the opportunity to express ourselves, and in other cases shutting down a conversation:

*“The words actually shape the experience. And I would say that it has the capacity to shape the experience far more than the experience has to shape the words because our comprehension of things comes through the language.”*

*(Participant 10, consumer)*

There was also an understanding that words can be felt differently and have different impacts on people depending on the context, including who says them and how,*”…words can be used in a wide variety of ways to free people, educate them, to harm them or to entrap them, depending on the person saying them” (Participant 13, consumer)*. What came across strongly from the participants was that in a clinical context, lived experience is shaped by more than just ‘words’. Participants discussed communication as an overarching concept to questions about language and labels, including the importance of clarity, intention and delivery in a care environment.

*“Well, there’s a lot to be said for delivery and tone. An awful lot to be said in how words land. You could probably take any of these words that I’ve even said, and if delivered in the right way, with some other gentler language around them, they land differently.”*

*(Participant 8, carer)*

*“How you talk to people if you’ve got good intentions, make them show. If you don’t, we can pick up on it. I think that is the sort of basis of the communication and the words. It’s not really the words. It’s the intention and communicating that…”*

*(Participant 4, consumer and carer)*

Participants raised concerns that bias, attitudes and assumptions could be ‘transferred’ between staff in handovers and notes, which impacted their overall experience of care. As this participant stated, *“Oh, you can tell. I mean, you can absolutely tell. You can tell how people are interacting with you has been influenced” (Participant 9, consumer)*. Supporting these assertions, participants who also worked in the health sector described how information is shared between shifts, in person and in written notes:

*“…working in the [job] in handover sessions, they will, instead of describing the situation they’ll just say ‘the patient is schizophrenic’ and from that you’re meant to draw what’s happened in the shift with that patient without them describing actually what has happened.”*

*(Participant 2, consumer)*

Demonstrating how interrelated the themes are, the problems associated with words having different meanings for different people, evolving language and ‘how the words land’ weave through the other themes and show the importance of maintaining a focus on individual experience.

#### 3.2.2. Feelings of Inclusion and Exclusion—Negative Experience and Memorable Connections

‘How the words land’ with the individual directly impacts feelings of inclusion and exclusion. While there was recognition that in general the language used to describe mental ill-health has improved to become more person-centred and inclusive, the participants gave many personal examples of exclusion, including in clinical contexts, the workplace, advocacy and community organisations, and the community more broadly.

*“In the environment of mental health, [language] can often offer hope and encouragement to people, and through exclusion by using certain terminology, it can often stigmatise people and lead to worse stigmatisation and also affect their condition by significantly deteriorating it.”*

*(Participant 2, consumer)*

When discussing feelings of inclusion, participants talked about feeling welcome, understood, accepted, connected and validated. Participants provided stories of ‘memorable connections’ where positive inclusive experiences gave a sense of hope and purpose to a personal recovery journey. One felt *“…we give so much away with our language, and those interactions I think, more often than not, can really promote dreams or they can absolutely squash them” (Participant 9, consumer)*. In addition, ‘memorable connections’ reflected the times when people were recognised and treated as a whole person, with a wealth of lived experience, not a collection of labels and behaviours:

*“Person-centred language I find is inclusive and sees the whole picture. Again there’s, you know, not person-centred language where it’s just about the label or it’s about the box, it’s about the problem presented as opposed to the whole person.”*

*(Participant 15, consumer and carer)*

Carer participants were particularly conscious about using language that included the person they care for, and faced challenges being able to speak for and about their loved one due to frustrations with privacy and exclusion. Carers said they bridged communication between services and the person they care for, navigating both personal and medicalised language:

*“…in a meeting with a psychiatrist in an area mental health service… so I know to step into that arena and have a productive discussion, like I said, one adopts the language, terminology, labels if you will, that are used within that setting. Do I feel good about that? No, but as I said, it almost becomes a shorthand for a productive discussion.”*

*(Participant 8, carer)*

Carer participants also acknowledged the importance of considering the wishes of the people they care for when speaking to others:

*“I’m constantly having to carefully pick and choose my language as you can tell by the way I’m talking in this interview to challenge those world views, but in bridging ways and um…safe and comfortable ways.”*

*(Participant 14, carer)*

Consumer and carer participants linked stigma and shame to feelings of exclusion, describing these as a dismissal of lived experience, which can lead to withdrawal and disconnection. Many felt disempowered by low expectations for people with mental ill-health and the associated loss of hope. For example, *“simplistic views of the people that have mental illness” (Participant 9, consumer)*, often reinforced by diagnosis: *“to tell people that you should lower your life expectations, that we know what your outcome will be” (Participant 13, consumer)*. Participants also talked about the enduring negative impact the words of others can have on individual journeys:

*“And throw in such unhelpful words that just cause damage for the whole journey…There’s no way to repair that, it’s not, like if it’s a one-time journey you’re lucky but like if it’s a teacher it’s a 12 month journey, if it’s a nurse in a unit then it’s every time you’re back in that unit.”*

*(Participant 15, consumer and carer)*

Similarly, they described difficulty speaking up and how feeling devalued and vulnerable excluded them from their own treatment, becoming passive recipients rather than feeling empowered in their own recovery. Staff attitudes and language were often perceived as reinforcing power imbalances, especially when already feeling vulnerable; one participant commented, “*I noticed that I was every psychiatrist’s favourite patient until I started asking questions about my medication and then I could see I was ruffling feathers” (Participant 13, consumer)*. Conversely, another said, *“I believe if you’re in hospital there’s no language that is powerful for me in that I can hardly speak to anybody because I feel so powerless” (Participant 4, consumer and carer)*. Some participants described similar experiences where power imbalances impact participation in workplace and advocacy, including the ability for a carer to advocate for the person they care for, *“*…*you don’t want to call them out on it when you’re in a meeting, because you’ve got to keep people on side, and aligned with what is best, hopefully, for the person they’re treating” (Participant 8, carer).*

Participants worried that inclusion efforts can exclude people who do not fit the group criteria, whether by diagnosis or identity. ‘Not fitting the box’ was a term used by participants to describe exclusion, and their examples ranged across lifetime and in clinical services, advocacy and community environments.

*“I just wonder whether the use of language which is meant to include actually does include or whether it’s actually excluding and isolating and putting a greater divide I guess…”*

*(Participant 15, consumer and carer)*

*“…who is part of the discussion, whose identity is validated, who’s told no, no, your experience is not enough to be lived experience, you’re not part of this group. And that has an emotional impact on people and a social impact on people.”*

*(Participant 7, consumer)*

Participants described how feeling excluded, being denied services or told they didn’t meet the criteria made reaching out for help difficult. One participant summed up how important language is to preserve connection when experiencing a ‘revolving door’ of services as *“the more times the door gets shut the more important the language used is…” (Participant 15, consumer and carer)*.

#### 3.2.3. Labels and How They Land

Labels were seen to shape group membership and influence feelings of exclusion, identity and self-worth: *“I think the less likely you are to fit into the box the more likely they are to shut the lid on the box” (Participant 15, consumer and carer)*. Participants highlighted additional issues with labels and how they land, including ‘othering’ language felt by many as exclusion; questioned if the search for diagnostic labels may *“limit curiosity” (Participant 10, consumer)* about individual needs, and make people feel broken:

*“And the inference, and I think it’s a reasonable inference that a vulnerable patient like me or somebody else would make is that…you are describing something that is broken in me and these treatments are targeted treatments that will fix the broken thing.”*

*(Participant 13, consumer)*

In the same way, participants described how feeling excluded had prevented them from seeking help and support; some participants highlighted that because they did not meet the criteria for a label, they were unable to access the services or help they required:

*“There is a big difference between how we see ourselves as not enough and how the sector might see us as not enough. Like for example as part of my lived experience, I had an eating disorder but I wasn’t at a low enough weight to get specialist eating disorder care and so to me, internally that just solidifies that I’m not worthy of care or I’m not sick enough to warrant care, even though my experience was really impacting my ability to live in the world…My eating disorder behaviours got pretty ingrained, because I wasn’t getting any help.”*

*(Participant 7, consumer)*

Many participants referred to labels as a double-edged sword. On the one hand, participants noted appreciating the benefits of labels, for example, providing access to services and support. On the other hand, participants expressed a range of scenarios where labels increased stigma and exclusion: *“I like them but I also hate them. I think they open doors but I also think they close doors very quickly” (Participant 15, consumer and carer)*, similarly:

*“…so if some individual is not coping particularly well, and if they happen to get the label of mental illness…You’ll be stigmatised. Anything you do to defend yourself will be just your mental illness.”*

*(Participant 6, consumer)*

Some participants believed that labels were helpful in enabling shared understanding with medical professionals and navigating treatment: *“I think they’re useful in setting a scene and a context in a therapeutic environment” (Participant 9, consumer)*. Many participants used diagnostic labels to research mental health conditions and improve their own knowledge, including understanding behaviours and past experiences, and even acquire a sense of relief and hope:

*“[They] gave me hope that that diagnosis might be a key to the room of the library in which I could learn more about myself and learn more about how my brain operated differently to other people’s brains.”*

*(Participant 12, consumer and carer)*

Participants also discussed the benefits of labels to share information without going into detail about personal experiences, “…when we have labels that are common and commonly understood, labels are terrific as short-hands for longer pieces of understood information” (Participant 12, consumer and carer). However, the possible misunderstanding of using diagnostic labels in this way was also raised as a problem. One participant described finding labels useful in social situations but wanted more descriptive ways to discuss their experience in a clinical setting. Whereas other participants felt that labels had a place in a medical setting but could lead to misunderstanding in social settings: *“definitely not socially. I would much rather take the labels off things and deal with people as people” (Participant 10, consumer)*.

Participants were concerned that having more than one label can result in some issues being overlooked: *“I also think if there’s multiple labels one often carries the power with the rest being hidden in shadows*…*” (Participant 15, consumer and carer)*. Several participants highlighted issues when people can believe in, or hold on too tightly to, a diagnostic label, causing harm. One participant remarked on being diagnosed as:

*“Pretty awful…whatever your hopes and dreams are for your life, uh-oh, you’ve got this thing. You know, it felt pretty much like a door closing…a death sentence, pretty much, because I knew the incurability of all that stuff.”*

*(Participant 4, consumer and carer)*

When discussing labels, participants also spoke about the common terms used to describe people within the mental health sector, such as ‘consumer’, ‘client’, ‘patient’ and ‘carer’. Participants articulated various perceptions and concerns about these labels based on personal experiences, also acknowledging that context makes a difference:

*“If we’re arguing about, you know, whether there’ll ever be a bigger slice of the pie for health in the total overall budget delivered by the treasurer, then the word that’s used to greater effect is consumers. If you’re talking about your own personal experience when you’re talking to your GP, you refer to yourself as their patient.”*

*(Participant 12, consumer and carer)*

Conversations about systemic terms, particularly ‘consumer’, illustrate that words mean different things to different people. For example, two participants expressed opposite views when describing what the term consumer means to them:

*“Oh, I hate it, because of the passivity of it, like you are the one, the sitting duck that all you can do is wait for people to help you out because you consume stuff, but you produce nothing. You’re like the hungry caterpillar, you know…”*

*(Participant 4, consumer and carer)*

*“It’s a more powerful term because the consumer is the person who takes and consumes the thing. So it puts you in a little bit of a position of control.”*

*(Participant 10, consumer)*

Importantly, the terms used by the mental health sector to describe people seeking services were not used by the people themselves—*“I’ve never seen myself as a consumer. I see myself as [first name]. They haven’t quite figured out what to call us, I think” (Participant 9, consumer)*. The terms ‘lived’ or ‘living’ experience were noted as more accepted, appropriate and respectful. Participants described ‘lived experience’ as an inclusive term, subject to less stigma than other systemic labels and a term people included in ‘what we call ourselves’ (possibly as part of their identity). However, one observation highlighted that the term ‘lived experience’, intended to be an overarching label that embraces everybody with experience of mental ill-health, is often clarified with a diagnostic label to aid understanding, and potentially sectionalise the lived experience community:

*“…lived experience of depression, or a lived experience of disordered eating. And I recognise that there’s labels in those lived experiences…I didn’t realise that I was doing that. But saying labels are bad and then using them because they’re easy. Interesting.”*

*(Participant 7, consumer)*

#### 3.2.4. Identity and Connection

While not everyone wished to identify with the labels created by the mental health sector, participants were sometimes prepared to adopt terms as part of their identity or as ways of describing themselves to others. Participants discussed the importance of connection to others with shared experiences and acknowledged that sometimes this comes as a benefit with a diagnostic label, *“I think people find their people. Yeah, I mean I think they would potentially find them without labels but I think labels makes it a whole lot safer*…*” (Participant 15, consumer and carer)*. For some participants, even with a complex relationship to diagnosis, having a diagnostic label provided opportunity for connection, which can aid personal recovery, but also came with strong questions about identity.

*“…as soon as I heard the word bipolar, I felt like I was allowed to now belong in a group of people who had really experienced some quite extreme states…that word bipolar then is a way for me, a touchpoint for me to connect with other people to have these conversations and it’s been really freeing. That’s been the therapy I needed.”*

*(Participant 13, consumer)*

*“I’ve noticed more and more that people introduce themselves to you as their diagnosis…as an example, 20 years ago, ‘G’day, mate, I’m [first name].’ Now it’ll be, ‘I’m [first name], and I’ve got complex PTSD.’…is that a good thing or a bad thing that people are willing to talk about it more or is it they’ve been constantly, had labels thrown at them that it’s now become part of their identity?”*

*(Participant 9, consumer)*

Carer participants had unified thoughts about the term ‘carer’ and their connection with the term as part of their identity. However, they observed that the carer identity is complicated by opinions of the word ‘carer’ in wider mental health advocacy and systematic circles, where using the term is often discouraged due to associations with uncomfortable power dynamics. There was a feeling among participants that terms like ‘family supporter’ do not reflect their role and responsibilities:

*“The [carer] label can at least provide some kind of social validation for what you’re doing with your life, and it’s usually a sense of failed life…if that’s taken away without replacing it, with strength based either structures, opportunities or other labels, I think it’s really problematic.”*

*(Participant 14, carer)*

Many of the carers told us that they identified as a carer because it was required by the system, *“I never used to refer to myself as a carer…until I realised quite some time ago that using some of these terms was a shorthand to a discussion” (Participant 8, carer)*. There was also a sense of loss associated with the carer identity, with one describing it as a *“robbed role” (Participant 15, consumer and carer)* where other identities such as ‘Mum’ and ‘family’ are lost:

*“I gave up my job as well to take on a more intensive carer role. You know, like so I’ve lost my work identity, I’ve lost finances, I’m now thrown back into a role and having a family member unwell…”*

*(Participant 3, carer)*

When speaking about language evolving around the carer label, one participant described how they experienced distress when told at a training course the term ‘carer’ should no longer be used. A significant part of their identity and experience was lost and invalidated at that moment.

As with the carer conversation above, what made it clear that some people had connections to a label as part of their identity was their experience when the label was taken away. These experiences ranged across hope for the future, goals being altered by diagnosis, impact of others on an individual’s journey, and terms being changed due to language evolution.

*“I think that was a legitimate dream of his to do that, that interaction with Nurse Ratched was just, “You’re not capable,” and therefore it just completely and utterly destroyed his day and his week and everything.”*

*(Participant 9, consumer)*

*“…your new language now actually seems a little bit strange and confusing and it actually doesn’t articulate what we’ve spent years accepting […] and the [confusion causes anxiety and distress] because [they are] back at that what am I?”*

*(Participant 15, consumer and carer)*

A similar experience of loss was described by participants who were told they did ‘not fit the box’ or were ‘not enough’ to receive a label and were therefore denied the opportunity to seek support, self-understanding or connection that participants told us was important to identity and recovery:

“Even if I could be told, ‘Yeah, look, I don’t think there is a diagnosable condition, maybe you need some other sorts of supports.’ That would be helpful. So it left me hanging and wondering, well what the f*** do I do now?”

*(Participant 10, consumer)*

When discussing how language evolves, participants gave examples of derogatory words that have been replaced over time. Several participants talked about feeling empowered to create or reclaim their own labels, and the importance of these labels to their identity:

*“The labels that are useful and powerful are the ones that people choose for themselves. I don’t think anyone has the right to be putting labels on anybody else that they don’t want. That’s the whole Mad Movement that’s happening. People reclaiming madness as a label for themselves, when previously they were labelled mad by someone else in a negative way. I don’t think we have a right to tell anyone how they identify their experience.”*

*(Participant 7, consumer)*

While the ability to reclaim past derogatory labels was seen positively by participants, there was an acknowledgment that, because words mean different things to different people, while some people find these new uses acceptable, the words can remain problematic for others.

#### 3.2.5. Evolving Language and Labels—Who Decides?

These examples of changing labels illustrate the dynamics of evolving language, which impacts how people can describe their experience. Participants expressed concerns relating to decisions about language changes and who decides what words are in and out. Participants noted that seemingly arbitrary language changes introduced by organisations can sometimes get it wrong; contribute to communication difficulties and exclude or dismiss lived experience. For example, a participant raised concerns about the ‘sanitisation’ of suicide, feeling that in following new guidelines, expression may be restricted:

*“It started with you can’t say the word committed suicide because that’s really upsetting for some people…We’ve gone from this offensive use of language relating to suicide, to this no, no, no, you can’t basically say anything and that just makes people too afraid to say anything at all then for fear of saying the wrong thing.”*

*(Participant 7, consumer)*

In our interviews, participants talked about the mechanisms for disseminating and perpetuating the language and labels associated with mental ill-health, including the Diagnostic and Statistical Manual of Mental Disorders (DSM-5) [39], language guides, media and education; acknowledging both positive changes and highlighting concerns. When referring to the classifications in the DSM-5, participants acknowledged the inclusion of new understanding and that as language and labels evolve, people with different experiences can feel safe to express their feelings to professionals. Conversely, participants also questioned its validity:

*“…the labels, in my mind, the DSM, look it’s a nonsense. There’s no science behind it at all. It’s all put together by a committee. […] So the labels are not helpful…They won’t help you overcome the crisis that you’re in.”*

*(Participant 6, consumer)*

There was a consensus that language guidelines were beneficial to remove stigmatising labels or terms and can be helpful in moving forward; but participants also thought that guidelines miss the point of individual experience:

*“I don’t think there should be a list of what’s appropriate or inappropriate because we’re all different and creating a list, it just assumes that we’re all the same, and we’re all happy to hear things.”*

*(Participant 7, consumer)*

Several participants raised questions about the number of language guidelines and whether they restrict what people can say, placing extra pressure on mental health practitioners: *“They’re hypersensitive to language now*…*instead of focusing on the person in front of you that needs your support, you’re worried about saying the wrong thing” (Participant 7, consumer)*.

#### 3.2.6. Doing Things Differently—Understanding the Person

As noted across other themes, participants identified negative and positive experiences associated with language and labels. Behind these comments was an acknowledgement that it is important to treat everyone as an individual and not impose a one-size-fits-all approach. Participants suggested several practical ways communication, language and labels could take the individual into account, including moving away from labels, viewing a person’s health holistically, and the power of positive words:

*“Whatever the person is comfortable with and exploring what that looks like…maybe it’s actually not about labels at all, maybe it’s just about the personal experience. Tell me where you’re at regardless of labels. Maybe the understanding isn’t in understanding the label, maybe it’s understanding the person.”*

*(Participant 15, consumer and carer)*

Participants noted a tendency to treat people the same way in clinical settings, based on assumptions and said they really wanted to be understood as an individual, which includes an understanding of their lived experience. Taking the time to find out about the person and consider ‘how the words land’ with the individual establishes trust, rapport and connection and enables people to engage: *“If the health professionals had more insight to each person, …then they would get better outcomes, like better engagement” (Participant 11, consumer and carer)*. One participant used the term *“compassionate curiosity”* describing this as being:

*“not closed off and you’re not saying these are the boundaries. You’re saying, let’s explore that as a dialogue…from that lens of, I care about you and so we’re having this conversation because I care.”*

*(Participant 7, consumer)*

Participants also observed that in some circumstances professionals adapt their communication for people with specific needs, such as people who are neurodiverse or have intellectual disabilities, but that these adjustments are not always extended to people with mental ill-health: *“…sometimes you think, well that special effort could be made with everyone, actually” (Participant 8, carer)*. One participant summed up a genuine intention to be inclusive as *“inclusive language…it’s more about an inclusive aura I guess” (Participant 15, consumer and carer)*.

Participants emphasised the importance of allowing individuals to choose their own labels and how they would like to be described, *“I wonder how much better our efforts would be if we could just say alright, you choose who you want to be called. You want to be a client? Sure, you can be a client, and then just moved on” (Participant 7, consumer)*. Some participants talked about new or creative ways to describe experiences, rather than relying on a label: *“Inside and outside mental health, that we are all really different…so perhaps some extra tools would be useful for us to describe ourselves” (Participant 13, consumer)*. Many participants felt that mental health labels were too restrictive, keeping people contained in a box, not describing them holistically; and highlighted the need to change the view of the mind as separate from the body. Underlying these comments was the negative connotation of the word ‘mental’ in ‘mental health’ and agreement that this label itself creates stigma, othering and exclusion:

*“It’s like, there’s something wrong with your brain. So, if you get your brain fixed, you’ll be fine, but to me mental health means a much more, it’s a society thing, and it’s a community development thing, and it’s what’s available in your life, how you’re educated, what type of experience you’ve had, like trauma, and all that, early childhood stuff…It’s not just, there’s something wrong with your brain.”*

*(Participant 4, consumer and carer)*

Instead, participants spoke about normalising how we talk about mental health and equating it to physical health. Several participants stated that mental illness should be viewed as a medical condition, like any other illness: *“In both instances nobody voluntarily signs up for those things and I would like to see them treated the same, given the same respect and the same sympathy” (Participant 1, carer)*. Alternative suggestions for mental ill-health were words like ‘distress’, ‘trauma’ and ‘mood health’:

*“mental illness is psychosocial as well as biological and by using words like trauma and distress…it helps reinclude people who are mentally ill (supposedly) back in the group and it relocates the problem back with their environment.”*

*(Participant 13, consumer)*

When discussing inclusion, participants highlighted the power of positive words to provide safety and a sense of hope for the future. Participants also suggested exploring words a person is comfortable or uncomfortable with and having an open conversation about language to establish these boundaries, *“if you make a mistake, you say, sorry, I don’t know which words make you feel comfortable…it’s that respectful thing of asking, not assuming” (Participant 4, consumer and carer)*. However, participants acknowledged that changing language and attitudes are linked, and that influencing change in a clinical setting may somewhat rely on peer workers and others:

*“people with lived experience working with those training in different roles across the sector…to say it straight…what is helpful language or helpful ways of communicating. And again, this takes in the aspect of delivery and tone.”*

*(Participant 8, carer)*

## 4. Discussion

Through a series of qualitative interviews (*n* = 15), the current study explored the use of language and labels within the mental health sector and its impact on people with lived experience of mental ill-health. A key finding to emerge from this research is that words and their delivery can have opposing influences on the individual, impacting feelings of inclusion or exclusion, creating hope or withdrawal, providing or robbing identity and contributing to holistic well-being and recovery or disconnection. Acknowledging and respecting the individual, their unique journey and lived experience is especially important in an environment where language is constantly evolving, and everyone has a personal perception of the language and labels used in the mental health sector. We found that experiences of the use of language and labels were complex and layered, with overlap, ambiguity and blurred edges between the six themes of ‘how the words land’, feelings of inclusion and exclusion, labels and how they land, identity and connection, language evolves—who decides? and doing things differently—understanding the person. Importantly, this finding suggests that fixed language rules in health care settings may not be sufficient to address stigma and exclusion, and that the effects of language are influenced by context and culture, and individual preference.

Many negative experiences of language and labels described by our participants appear to be driven by stigma and power dynamics, known to cause harm and distress, including reducing self-esteem, discouraging help-seeking and reducing control over health-promoting behaviours [40,41]. This highlights how language, labels and communication shape self-perception and influence pursuit of personal goals, described by Corrigan et al. [42] as the “why try” effect. Participants also described manifestations of stigma through the perception and discrimination of people with diagnostic labels of mental illness [43,44,45]; stereotypes and biases that made people feel judged, dismissed and broken [44,46,47]; impacts on experience of care and health services [48,49,50]; and the influence of diagnostic and other labels on identity (loss of identity) and personal recovery [44,51].

Participants in this study also talked about the power of positive words to provide a sense of hope. For example, rather than making people feel powerless and dehumanised, helpful language can provide validation and hope [44,46,47,49]. Murphy et al. [52] provide evidence of the importance of hope to the recovery journey and the alignment of hope with trust, safety and connection, which were also mentioned by our participants in relation to the power of positive words.

Alongside the power of positive words, our participants provided recommendations for improvements in language, including moving away from labels, and focusing on individual experience, particularly in clinical settings. Newer approaches to framing and understanding mental distress, such as the Power Meaning Threat Framework [53], may influence future use of diagnostic labels and address stigma and the other negative effects participants described. However, such advice, including the use of person-first language, remains poorly implemented by health care practitioners [54,55]. At a broader policy and social level, other ideas range across alternative viewpoints on mental health diagnosis [44,56]; developing person-first and identity-first language and seeking personal preferences from individuals [57]; and creating language guidelines to raise awareness about the impact of stigma and promote change [58,59]. These suggestions are supported by the findings from quantitative surveys about the power of the use of person-first language to reduce stigma and improve attitudes in the community [60]. Similar to the concerns expressed by our participants, the argument for language guides is tempered by Corrigan’s [61] comments about “word police” stunting dialogue by imposing the same words on everyone, when, as our research shows, there is no consensus on preferred language. From a linguistic perspective, Galasinski [62,63] highlights the complications associated with language, personal perceptions and experiences, also arguing for more considered professional communication skills and motivations.

Lived experiences of language and labels could be improved if people were respected as individuals when seeking support. This was expressed as a desire for clinicians and others to see the individual person, be curious about them, apply more reflection to their communication to improve outcomes [46] and improve quality of communication and person-centred care [52,61,62,63]. Richards [64] calls on other health professionals to consider the language and communication they use, commenting on the dual impacts of language and the capacity for negative words to stigmatise and isolate people while positive words can demonstrate hope and empathy, also recognising that individuals have their own language preferences. Similarly, research associated with health records suggests that to provide person-centred care, clinicians should pay attention to language, particularly language perceived as stigmatising, consider how their words might be interpreted by other health professionals, and what impact this might have on patient care [48,49,50]. These recommendations parallel our central theme ‘how the words land’.

### 4.1. Practical Implications and Future Research

Understanding lived experience perspectives on language has implications in multiple settings, including clinical and community mental health, the media, and the community more broadly. These findings advance our understanding of the impact of language and labels on people with lived experience of mental ill-health. This research could be used to inform media campaigns, policy and guidance on appropriate language use, such as the Mindframe media guidelines [65]. Specifically, future guidelines may consider a more nuanced approach to ensure the broadest possible reach and avoid unintended exclusion or complicated communication. They could include non-prescriptive language recommendations and guidance on open conversations to validate personal preferences and boundaries to language and words.

Recognising that people’s lived experience influences how they might perceive and respond to certain words and communication styles can also advance research into provider-patient communication and support health care workforce training in improved communication styles [66,67]. This may also provide justification for lived experience peer workers to assist with training new professionals.

The suggestions provided by participants with regard to personalised communications and changes to language and labels could be used in future research to establish the relationship between ‘how the words land’ and outcomes for people with mental ill-health and their help-seeking behaviour.

### 4.2. Limitations

Interviews were conducted in English, which may have been a barrier for people from non-English speaking backgrounds. Whilst people with other cultural backgrounds and languages were not excluded, no participants provided an explicit CALD or First Nations perspective to the research or alternative non-Western understandings. Further research with these communities and other priority groups, for example young people and the LGBTQIA+ community, could provide different perspectives on language and communication. As is typical of qualitative mental health research, the participants in this study were primarily women [68] aged 35 years or over, and were an engaged, self-selected group who were able to reflect on their experiences and the impact of language and labels on these experiences. Therefore, the findings may not represent the perspectives of other genders, younger age groups, or those less comfortable discussing these issues. During the interviews, the co-researchers disclosed their own lived experience; while this was intended to build trust and rapport with participants, it is possible that social desirability may have influenced the stories that participants shared. As participants were recruited through established lived experience networks based in the ACT and Victoria, these results may not reflect experiences in other states/territories, or include experiences across rural, remote and metropolitan areas. Finally, we only collected basic demographic data; thus, it is possible that other factors (e.g., occupation) may have altered our interpretation of the results.

## 5. Conclusions

Lived experience participants provided rich insights into how language, labels and communication more broadly shape the experience of people with mental ill-health. Their main message to improve communication and outcomes was to centre the individual, take care to understand their unique experience and what this means for the words and language they choose to use. Words are powerful and so is the way they are delivered and received. It is important to consider ‘how the words land’ with the individual, alongside what this may mean for broader attitudes, beliefs and social inclusion. While participants acknowledged that labels can sometimes be useful, they expressed a strong desire to be seen and understood as a whole person, not just a collection of labels and behaviours. Language and communication that acknowledge the individual also provide connection, engagement and hope for personal recovery.

## Figures and Tables

**Table 1:** Theme definitions and sub-themes.

Themes	Description	Sub-Themes
How the words land	Central theme characterising the impact of language and labels on the individual. This theme binds the overlap between how an individual might respond to words based on their own experiences, with the wider/external influences on language, including stigma, evolving language, different interpretations of words, context and delivery.	Everyone is an individual
		Language evolves over time
		Words mean different things to different people/contexts
		Attitude and intention—delivery and tone
		The power of language
Feelings of inclusion and exclusion—negative experience and memorable connections	Experiences that described the positive and negative impact of language and labels. These are individual experiences that describe how words impact people, and provide examples of the variety of experiences from hope to disconnection. Here participants again reflected on words meaning different things to different people and depending on context, which therefore change how individuals respond. They also talked again about attitude and how this lends to the interpretation and response to language and labels.	The power of positive/negative language
		Memorable connections
		Concerns about exclusion—what does it feel like
		Expectations for the future
		Stigma and shame
		Perceptions of power
		What does inclusion feel like—validation, acceptance, engagement, hope
		Withdrawal and disconnection
Labels and how they land	Provides the perspective of the impact of both diagnostic and systemic language, e.g., consumer; and the mixed feelings participants had about labels—providing information about why they are useful, and why labels have negative impacts on people, including exclusion.	Labels (words) mean different things to different people
		Labels create inclusion and others (exclusion)
		Usefulness depends on context
		Labels are a double-edged sword—people hate them and see benefits
Identity and connection	Complicated relationships with labels, demonstrating that for some people identity forms around both diagnostic and systemic labels, and that these identities can help people make connections, aid personal recovery, and help describe experience. Loss was also associated with experiences of labels and identity, which relates to exclusion and evolution of language. Overlapping with words meaning different things to different people, for example, reclaiming the word ‘mad’.	Self-worth and identity
		Finding your people—belonging/connection and understanding
		Dreams/goals dismissed by others (loss)
		Loss of identity
		Not fitting the box—help seeking
		What we call ourselves—choosing our own labels
Evolving language and labels—who decides?	Questions about who makes the decisions to use language in a certain way, and issues arising from language guidelines.	Impact on lived experience
		Language guides
Doing things differently—understanding the person	Participants suggested improvements to communication, language and labels. Treating everyone as an individual, seeing the whole person and exploring their experience and the words they find acceptable. Includes ideas of moving away from labels and alternative views of mental health.	Holistic health
		Understand the individual—how the words land
		Power of positive words

**Table 2:** Participant demographics from brief demographic survey.

Participant Demographic		*n*	%
Gender	Woman	12	80.0
	Man	2	13.3
	Different term	1	6.7
Age (years)	35–44	5	33.3
	45–54	2	13.3
	55–64	4	26.7
	65+	4	26.7
Lived experience	Consumer	7	46.7
	Carer	4	26.7
	Both (consumer and carer)	4	26.7
State/Territory	Australian Capital Territory	6	40.0
	New South Wales	1	6.7
	Queensland	1	6.7
	Victoria	5	33.3
	Western Australia	2	13.3

Note: Age categories and State/Territory with no participants have been omitted from the table.

## Data Availability

The data that support the findings of this study are available on request from the corresponding author (M.B.). The data are not publicly available as they may contain information that could compromise the privacy of research participants.

## Supplementary Materials

The following supporting information can be downloaded at: https://www.mdpi.com/article/10.3390/ijerph23081032/s1. Supplementary Table S1: Initial codes reflecting concepts of mental health, lived experience, language, labels, communication and identity. Supplementary Table S2: Initial six themes and three overarching themes from team analysis. Supplementary Table S3: COREQ Checklist. Ref. [69] was cited in the Supplementary Material, checklist available at: https://onlinelibrary.wiley.com/pb-assets/assets/17416612/COREQ_Checklist-1556513515737.pdf (accessed on 4 August 2026).

## Abbreviations

The following abbreviations are used in this manuscript:

ACACIA	The ACT Consumer and Carer Mental Health Research Unit
ANU	The Australian National University
ACT	Australian Capital Territory
ALIVE	The ALIVE National Centre for Mental Health Research Translation
RTA	Reflexive thematic analysis
