# Supplementary material for: It’s Not Really the Words; It’s How They Land: Lived Experience Perspectives on the Use of Language and Labels in Mental Health
Source: Int J Environ Res Public Health. 2026 Aug 7;23(8):1032. doi: 10.3390/ijerph23081032 (PMC13512354; doi:10.3390/ijerph23081032)
Supplement: Supplementary file 1 [file ijerph-23-01032-s001.zip › Supplementary Table S2.pdf]

Supplementary Table S2: Initial six themes and three overarching themes from team analysis

During initial theme development six themes were identified (numbered) containing three overarching themes (bold).

1	**People experiences are different** - what is lived experience, how is it encompassing, when we use it why are we just talking about LE rather then LE of different things.
2	The good the bad and the ugly of labels - includes identity as a sub theme. Words shape our experience, taking away a word dismisses the exp. Includes and excludes
3	**Language evolves over time** - not a static thing. Language should be diverse and encompass many things. Sometimes the evolution is good sometimes not.
4	It's not all about the words - tone matters, attitudes, behaviours, and things that come with the language. Delivery.
5	Impacts. What might actually happen as a result of the language we are using. Connects into impacts and power of connecting and excluding. Hope and power, stigmatising. **Power of positive and negative language**.
6	organisation/media - have tried to address these things however they are not thinking about language evolves and is not static. Language guides, attempt to provide some education however they can't be prescriptive. Impact on other people when decisions just get made.
