# Supplementary material for: It’s Not Really the Words; It’s How They Land: Lived Experience Perspectives on the Use of Language and Labels in Mental Health
Source: Int J Environ Res Public Health. 2026 Aug 7;23(8):1032. doi: 10.3390/ijerph23081032 (PMC13512354; doi:10.3390/ijerph23081032)
Supplement: Supplementary file 1 [file ijerph-23-01032-s001.zip › Supplementary Table S1.pdf]

Supplementary Table S1: Initial codes reflecting concepts of mental health, lived experience, language, labels, communication and identity

Concept	Category	Sub category	Codes
Mental Health	Perceptions of mental health		How we talk about mental health
			Mental health is separated from physical or general health
			People experience conditions differently
			Physical pain and emotional pain
Language	The power of language	Views on language as a concept	Words shape experience
			Words can be felt in different ways
			Language/words can provoke an emotional response
			How we think about language
			Words mean different things to different people/contexts
			Talking about lived experience
		Language evolves over time	Words mean different things to different people/contexts
			Inclusive language improvements
			Who is in charge of language changes and has it gone too far
			Change of understanding if labels/language are coopted by the system
		Education and media	Mental health literacy/education improves understanding
			Staff Education
			Stigma and shame perpetuated by media
			Language guides and standards
	The power of language - negative/excluding language	Concerns about exclusion - what does it feel like	Impact of disclosure of diagnosis
			Results of exclusion (impact of exclusion)
			Creating inclusive groups also creates "others"
			Excluded from system/care - not enough/don't fit the box
			Excluded from participation
			Personal feelings of exclusion - I am not enough/other/broken
			Language in our internal monologue
			Stigma and shame
			Falling back on self-reliance
		Homogenisation of language in mental health	What do the words mean to the individual - how do the words land
		Expectations of the future	Low expectations for people that have mental illness
			Expectations of recovery - loss of hope
			Dreams/goals dismissed or discouraged by others (loss associated with this)
		Response to negative words and language	Feeling dismissed/disrespected causes shut-down/disengagement/put up a wall
			Being spoken to and treated in the wrong way is crushing
			Unexpected response when lived experience is discounted
			People may not react to language as expected
		What does negative language look and feel like	Negative language/tone discounts lived experience
			Negative language/tone is disrespectful
			Negative language can damage the journey
			Negative language – name the diagnosis/condition focus
	The power of language - positive/including language	What does positive language look and feel like	Positive language provides hope for the future
			Positive language – name with mental illness
			Positive language is respectful and supportive
			Positive language demonstrates acceptance
			Positive language - uses normal/familiar words

			Positive language helps people feel safe to speak/engage
		What does inclusion feel like	Connected, engaged and able to speak
			Validation and acceptance
			Recognises experience
			Hope for the future
Identity	Self-understanding, identity and loss of identity	Label as identity	Plans for the future/future identity as a label
			It's cool to have a label
			Connection, understanding and voice
			I am not defined by the condition/diagnosis/label
			Using a label is an easier way to communicate what is going on in some cases eg. Social situations
			Negative views of self
			Let people choose what they would like to be called
			Inhabit a label for advocacy/system reform
		Loss of label or identity	Not enough to be given a label (don't fit the box) - what am I? Impact on self-understanding - dismisses experience
			Reluctant to own a label if my experience diminishes the experiences of others with the same label
			Changing labels can be confusing
			Changing language/labels is not easy for people who have gathered self-understanding or identity with other language
			Taking away the word dismisses the lived experience (loss)
		Loss of label or identity (carer)	Being a carer means other parts of your life/identity are taken away
			Describing the word carer as outdated language dismissed my role – taking away my identify and challenging my self-worth
Labels	The good the bad and the ugly of labels	Good things about labels - self-understanding and identity	Diagnostic labels - enable connection and understanding
			Diagnostic labels - useful for self-understanding/validation
			Label as identity
		Good things about labels	Diagnostic labels - useful in medical context
			Diagnostic labels - access to services/treatment
			Diagnostic labels - protection from discrimination
			Diagnostic labels - useful shorthand in social situations
			Labels provide a connection
			Labels provide a shared understanding
		Bad things about labels	Labels are a double edged sword - people hate them and see benefits
			Labels usefulness changes in different contexts
			Labels prevent curiosity and engagement with a person as an individual
			Negative response to labels
			Labels might need an explanation to provide context
			Using behaviour as a label
			Diagnostic labels - can be misunderstood
			Diagnostic labels - do not describe experience
			Diagnostic labels - make me feel broken
			Diagnostic labels - misappropriation/generic use is a problem
			Diagnostic labels - can become an a definition or an excuse
			Diagnostic labels - stigma and shame
			Lived experience as a label

		Ugly things about labels	Competition between consumers for most extreme label
			Labels cause assumptions, separation, difference and othering
			It's cool to have a label
		Words mean different things to different people	Comments on specific words/labels – consumer
			Comments on specific words/labels – client
			Comments on specific words/labels – patient
			Comments on specific words/labels – carer
			Comments on specific words/labels
Lived experience	Understanding the individual's lived experience	Carers' lived experiences	Care relationship
			Communication with and about the person cared for
			The carer role and identity (and label)
			Observations of exclusion of carer identity
			Everyone can be vulnerable
		Recognition of lived experience	Lived experience advocacy
			Peoples experiences of mental health concerns are all different – even if they have the same label
			Seeking to understand each person as an individual
			Too much attention on labels - extra tools to describe ourselves
			Valuing/accepting the individual and treating them as a person, not their mental illness
			Language based on factual information is more inclusive of experience
			Experience dismissed - opportunities for connection and support reduced
			Talking about symptoms as a way to describe experience
			Person-first People with lived experience of mental health condition
			Explore what words are OK as part of a conversation
			Don't treat everyone the same – make a special effort for everyone
			How we speak should resonate with the individual
			Can't just use learnt responses without understanding individual's experiences
			People's opinions/assumptions can dismiss lived experience
			Trauma and environment
Communication	Communication is critical	Communication with services - it's not all about words	Inclusive communication
			Actions speak louder than words
			How the words land
			Ability to participate (maybe more about experience)
			Service promotion
		Communication with services - staff attitudes and behaviours	Better ways to work together
			Communication is critical
			What is communicated between staff
			Perception of power
			Negative attitude conveyed to vulnerable person
			Attitude and intention (could be the same as assumptions?)
			Assumptions influence interaction (could be the same as attitude?)
			Good professional-client relationships have trust and rapport
			Impact of others - made to feel safe/understood
			Impact of others - causes harm and damages the journey

		Memorable connections/impact of others	Impact of others - wildly different approaches
			Memorable connections - make the world of difference (positive)
			Memorable connections - make the world of difference (negative)
		Alternative communication	Learn creative ways of expression from other fields eg. creative arts
